# Comparative genetic analysis of blood and semen samples in sperm donors from Hunan, China

**DOI:** 10.1080/07853890.2024.2447421

**Published:** 2025-01-06

**Authors:** Chuan Huang, Li-Ming Chu, Bo Liang, Hui-Lan Wu, Bai-Shun Li, Shuai Ren, Mei-Ling Hou, Hong-Chuan Nie, Ling-Yin Kong, Li-Qing Fan, Juan Du, Wen-Bing Zhu

**Affiliations:** a Clinical Research Center for Reproduction and Genetics in Hunan Province, Reproductive & Genetic Hospital of International Trust and Investment Corporation (CITIC)-Xiangya, Changsha, China; b NHC Key Laboratory of Human Stem Cell and Reproductive Engineering, School of Basic Medical Sciences, Central South University, Changsha, China; c Basecare Medical Device Co., Ltd, Suzhou, China; d State Key Laboratory of Microbial Metabolism, Joint International Research Laboratory of Metabolic and Developmental Sciences, School of Life Sciences and Biotechnology, Shanghai Jiao Tong University, Shanghai, China

**Keywords:** Sperm donor, genetic analysis, whole-exome sequencing, sperm, blood

## Abstract

**Objectives:**

At present, most genetic tests or carrier screening are performed with blood samples, and the known carrier rate of disease-causing variants is also derived from blood. For semen donors, what is really passed on to offspring is the pathogenic variant in their sperm. This study aimed to determine whether pathogenic variants identified in the sperm of young semen donors are also present in their blood, and whether matching results for blood are consistent with results for sperm.

**Methods:**

We included 40 paired sperm and blood samples from 40 qualified semen donors at the Hunan Province Human Sperm Bank of China. All samples underwent exome sequencing (ES) analysis, and the pathogenicity was assessed according to the American College of Medical Genetics (ACMG) guidelines. Scoring for sperm donation matching, which was based on gene scoring and variant scoring, was also used to assess the consistency of sperm and blood genetic test results.

**Results:**

A total of 108 pathogenic (P)/likely pathogenic (LP) variants in 82 genes were identified. The highest carrier had 7 variants, and there was also one donor did not carry any P/LP variant. On average, each donor carried 2.7 P/LP variants. Among all the P/LP variants, missense mutation was the dominant type and most of them were located in exonic regions. Chromosome 1 harboured the largest number of variants and no pathogenic copy number variants (CNV) was identified in semen donors. The P/LP variant of all the 40 semen donors was consistent by comparing sperm and blood. Except for one case that was slightly different, the rest simulated matching results for blood were all consistent with results for sperm.

**Conclusions:**

It is reasonable to choose either blood or sperm for genetic screening in semen donors.

## Introduction

The establishment of China’s first human sperm bank by Lu Hui-Lin and Lu Guang-Xiu in 1981 has led to the growth of 29 sperm banks across the country today [1]. Existing regulations mandate rigorous laboratory testing, including karyotype analyses, to exclude genetic disorders in potential donors [2,3]. However, these protocols are insufficient to meet the increasing need for comprehensive genetic screening. Karyotype analyses have limitations in detecting a wide range of hereditary risks, including both autosomal recessive and dominant conditions with complex inheritance patterns [4]. This situation highlights the necessity for improved genetic testing and evaluation protocols for prospective semen donors.

To date, genetic screening for semen donors has focused on specific disorders such as spinal muscular dystrophy, cystic fibrosis, thalassemia, Tay-Sachs disease, and fragile X syndrome, or has been limited to small expanded carrier screening (ECS) panels that cover only 46 disorders [5–8]. Considering the significant carrier rates for these conditions, there is a compelling argument for implementing broader genetic testing panels to enhance donor screening protocols.

Exome sequencing (ES) is a powerful and cost-effective tool for dissecting the genetic basis of diseases and traits that have proved to be intractable to conventional gene-discovery strategies [9]. ES is more effective than ECS in terms of both efficiency and accuracy, achieving a 30–40% rate in detecting pathogenic or likely pathogenic variants [10]. Sequencing of sperm from healthy men has identified clonal mosaic mutations that are different from those in blood and stable over time [11]. However, genetic screening in clinical practice generally is conducted using blood samples. To our knowledge, there is a lack of studies that compare the genetic analysis of blood and sperm samples from the same patients by ES, especially the comparison of pathogenic variants. We aimed to determine whether pathogenic variants identified in the sperm of semen donors are also present in their blood and whether matching results for blood are consistent with results for sperm. To this end, we conducted a prospective study of pathogenic variants in the blood and sperm of semen donors by ES combined with additional panels.

## Methods

### Study population and participants

From January 1, 2022, to May 1, 2022, 40 qualified semen donors were enrolled at the Hunan Province Human Sperm Bank of China. All donors signed informed consent forms during their first visit to the sperm bank, consenting to the use of their sperm and blood samples or data by the sperm bank for scientific research purposes. During the same period, two women seeking sperm donation for conception were randomly selected to participate in simulated matching tests (primarily using their ES test data), and they also signed informed consent. both sperm donors and patients were deidentified through the use of personal numerical identifiers instead of names. This study was conducted in accordance with the Declaration of Helsinki and was approved by the Institutional Review Board (IRB) of Central South University. The number for the approval was 2021-KT48.

### Criteria for semen donor screening in China

Table S1 illustrates the key guidelines and standards for sperm donor screening in China, and donors must meet this criteria [12,13].

### Sperm and blood samples

Sperm and blood samples were collected from 40 semen donors who ejaculated into a sterile container after abstaining for 2–7 days. The samples were assessed according to WHO recommendations [14], analysing volume, sperm concentration, morphology and motility based on WHO’s motility grades (PR, progressive sperm; NP, non-progressive sperm; and IM, immotile sperm). Blood samples of 1.5 mL were collected from each donor and stored in 2-mL cryopreservation tubes at −80 °C until needed.

### Genetic testing

Peripheral blood and sperm samples were collected from semen donors. The TIANamp Blood DNA Kit (TIANGEN) was used to extract blood genomic DNA (gDNA), and the nucleic acid extraction or purification reagent (Basecare, China) was used to extract sperm DNA. The SureSelect Human ALL Exon V6 (Agilent) kit was used for liquid capture hybridization, elution and library amplification, and magnetic beads purification to obtain the whole Exon library. The final library was sequenced at 150 bp in pair-ends by Novaseq 6000 (Illumina) platform. For *SMN1/2* and *CYP21A2*, which were limited by the ES assay and relatively prevalent in the Chinese population (spinal muscular atrophy, and congenital adrenal hyperplasia).

We additionally added panel assays. *CYP21A2* was detected using LR-PCR combined with NGS, and we designed a long fragment primer of specific full-length *CYP21A2* fragment for NGS with DA8600 platform. For gene copy number detection of *SMN1/2*, we used multiplex fluorescent PCR and capillary electrophoresis (ABI 3730XL), and LR-PCR combined with NGS was used to detect and validate 15 common *SMN1* mutations in the Chinese population.

### Data analysis

The sequencing data were aligned to GRCh37/hg19 reference genome (map reads to reference) by Sentieon^®^ software (San Jose, CA, USA). And then marking duplicates, base quality score recalibration (BQSR) and variant calling were processed. For the VCF files after variant identification, single nucleotide ­polymorphisms (SNPs) and indels were filtered and screened according to sequence depth and mutation quality. Annovar© (https://annovar.openbioinformatics.org/en/latest/) was used to annotate variants, ­including the population frequency database 1000 Genomes (https://www.internationalgenome.org/), gnomAD (https://gnomad.broadinstitute.org/), ExAC (https://gnomad.bro­ad­institute.org/), ESP. Provean© (https://www.jcvi.org/research/provean), Sift© (https://sift.bii.a-star.edu.sg/), PolyPhen-2© (http://genetics.bwh.harvard.edu/pph2/index.shtml), LRT© (https://sites.google.com/site/jpopgen/dbNSFP), Mut­ation­Taster© (https://www.mutationtaster.org/), Mutat­ionAssessor© (http://mutationassessor.org/r3/), FATHMM© (https://fathmm.biocompute.org.uk/fathmm-xf/), CADD© (https://cadd.gs.washington.edu/), VEST© (http://www.cravat.us/CRAVAT/), MetaSVM© (https://sites.google.com/site/jpo­pgen/dbNSFP), MetaLR© (https://sites.google.com/site/jpo­pgen/dbNSFP), M-CAP© (http://bejerano.stanford.edu/mcap/), and Revel© (https://sites.google.com/site/jpopgen/dbNSFP) were used for protein biological function prediction

Synonymous mutations and deep intronic variants were filtered out. Variants were classified according to ACMG guidelines as ‘pathogenic (P)’, ‘likely pathogenic (LP)’, ‘unknown clinical significance’, ‘likely benign’ and ‘benign’ [15]. Variants were also divided into three types: blood-detectable only (BDO), sperm-detectable only (SDO) and blood-sperm shared (BSS) [16]. Data were also used for CNV calling by Sentieon software, and the identified CNVs (>200kb) were filtered and annotated. CNVs with ≥50% reciprocal overlap are classified as the same CNV locus between sperm and blood [17]. CNV pathogenicity was assessed according to the ACMG guidelines [15].

As sperm parameters of 40 sperm donors follow markedly skewed (non-normal) distributions, unadjusted mean and median values, standard deviation (SD) and 5th to 95th percentiles were calculated for each variable. Paired *t*-test was used for paired samples by SPSS V26.0 software, which obtained a copyright license.

### Simulated matching test in artificial insemination by donor

A simulated matching test was conducted using two randomly selected women. The sperm donation matching process primarily involves gene and variant scoring. Gene scoring evaluates several factors: mode of inheritance, gene–disease association degree, age of onset and disease severity. Data for this evaluation are sourced from OMIM, Orphadata, DisGeNET and GenCC databases. These factors are assigned different weights in the scoring process. Variant evaluation follows ACMG guidelines. In the matching process, each woman is paired with 40 potential semen donors. Donors are categorized as ‘recommended’ or ‘not recommended’ based on the presence of pathogenic risk genes. Additionally, a matching score is calculated to facilitate the decision-making process.

## Results

### Clinical characteristics of semen donors

The clinical characteristics of all qualified semen donors are summarized in Supplementary Table S2. Meanwhile, the average semen volume among donors was 2.8 (2.0–4.8) [Median (5^th^–95^th^%)] mL, with mean sperm concentration and progressive motility levels of 63 (60–71) [Median (5^th^–95^th^ %] million/mL and 52 (50–54) [Median (5^th^–95^th^ %)], respectively.

### Consistent pathogenicity variant in sperm and blood

**Figure 1.**
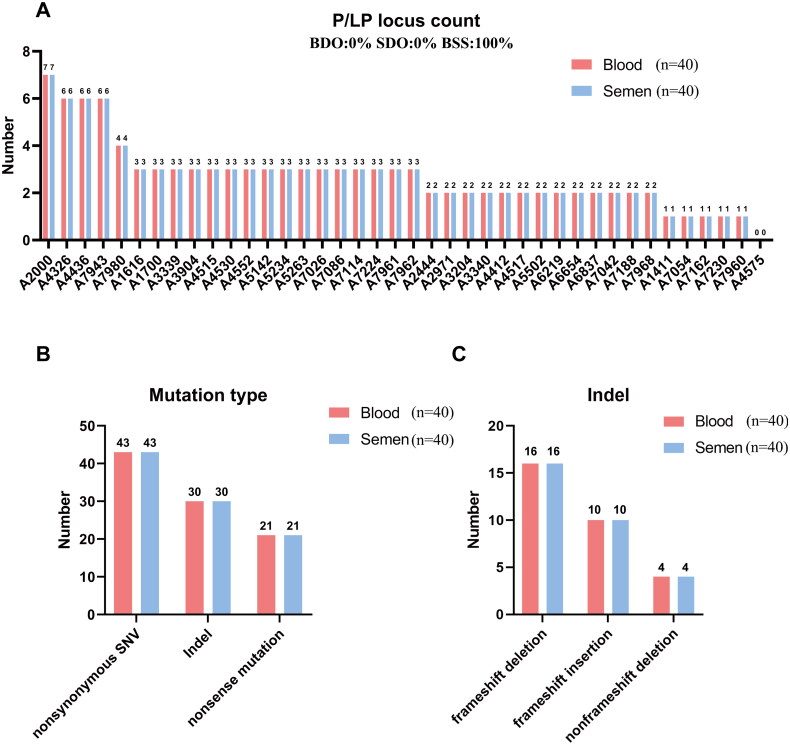
Comparison of mutation types between sperm and blood of sperm donors. (A) Comparison of the number of P/LP variants between sperm and blood of semen donors, the abscissa label is the sample number. BDO: blood-detectable only; SDO: sperm-detectable only; and BSS: blood-sperm shared (B) Comparison of mutation types (nonsense mutation, stoploss, splicing, synonymous/nonsynonymous SNV) between sperm and blood of sperm donors. (C) Indels (frameshift deletion/insertion, non-frameshift deletion/insertion) comparison between sperm and blood of sperm donors.

Figure 1 compares mutation types between sperm and blood of sperm donors, the results of the comparison analysis of sperm and blood samples from each donor revealed a high degree of concordance in the number of pathogenic or likely pathogenic (P/PL) variants (Figure 1(A)). As shown in Table S3 which compares of all the pathogenic and likely pathogenic SNVs between semen and blood, a total of 108 P/LP variants in 82 genes were identified, and the highest carrier had 7 variants, and there was also one donor did not carry any P/LP variant. All P/LP variants are present in both blood and sperm. On average, each donor carried 2.7 P/LP variants. Further, the proportion of mutation genes in sperm donors presented in Table 1, nine donors had P/LP variants (NM_004004.6: c.109G > A, c.235delC) in *GJB2* (Deafness, recessive autosomal 1 A), four donors each carried *CD36* (Platelet glycoprotein IV deficiency; NM_000072.3: c.329_330del, c.1227_1238del, c.1156C > T) and *DUOX2* (Thyroid dyshormonogenesis 6; NM_014080.4: c.1588A > T, c.2654G > A, c.1871delG, c.1588A > T) mutation, and three donors each carried *CFTR* (Cystic fibrosis; Congenital bilateral absence of vas deferens; NM_000492.4: c.1210-12T[5]/c.1210-34TG[12], c.1210-12T[5]/c.1210-34TG[13]) and *LIPH* (Hypotrichosis 7; NM_139248.3: c.736T > A, c.686_687insCCTGGCT, c.685_686insGTAGAACCCA) mutation (Table 1). As shown in Supplementary Table S3, the most frequent variant detected in semen donors was *GJB2* c.109G > A. The detailed all variants were shown in Supplementary Table S3 and S4.

**Table 1.** Genes with the highest proportion of mutations in sperm donors (genes with more than 2 cases).

Gene	Inheritance	Associated disease	Positive case	*N* = 40 Percentage(%)
Top 13 gene				
*GJB2*	AR	Deafness, autosomal recessive 1 A	9	22.5
*CD36*	AR	Platelet glycoprotein IV deficiency	4	10
*DUOX2*	AR	Thyroid dyshormonogenesis 6	4	10
*CFTR*	AR	Cystic fibrosis; Congenital bilateral absence of vas deferens	3	7.5
*LIPH*	AR	Hypotrichosis 7	3	7.5
*ABCA4*	AR	Stargardt disease 1	2	5
*ABCG5*	AR	Sitosterolemia 2	2	5
*AGL*	AR	Glycogen storage disease IIIa; Glycogen storage disease IIIb	2	5
*AIRE*	AR/AD	Autoimmune polyendocrinopathy syndrome, type I, with or without reversible metaphyseal dysplasia	2	5
*GALC*	AR	Krabbe disease	2	5
*HFE*	AR	Hemochromatosis 1	2	5
*SERPINB7*	AR	Palmoplantar keratoderma, Nagashima type	2	5
*UGT1A1*	AR	Gilbert syndrome	2	5

Note: AD, autosomal dominant; AR, autosomal recessive; “+” for consistent and “-” for inconsistent with the results between semen and blood.

Unexpectedly, two semen donors were found to harbor autosomal dominant (AD) mutations, which were consistent in both sperm and blood samples, one donor had a likely pathogenic (LP) mutation in the TPM2 gene (NM_003289.3: c.564-1G > A), potentially causing distal arthrogryposis (MIM #108120) or nemaline myopathy 4 (MIM #609285); and the other with *ACTN1* (NM_001102.4: c.1348C > T, LP), which would cause bleeding disorder (platelet-type, MIM #615193). However, these two semen donors did not exhibit any clinical manifestations of the respective diseases.

Analysis revealed that sperm samples exhibited a slightly higher frequency of certain types of non-pathogenic variants compared to blood samples. The detection threshold for variant allele frequency (VAF) in our methodology was 0.05. Variants specific to sperm samples are detailed in Supplementary Table S5. Moreover, Supplementary Figure S1, Supplementary Figure S2 visualizes these variants included benign, likely benign and VUS (variant of uncertain significance). Benign and likely benign variant in the sperm and blood of 40 semen donors showed that SDO variant number was significantly higher than that of BDO in the types of variants which were near splicing site, synonymous SNV, nonsynonymous SNV, non-frameshift deletion, frameshift deletion, frameshift insertion, and non-frameshift insertion. And for variant of uncertain significance, variant number in 3’UTR was more numerous in SDO than that in BDO. However, the number of unique non-pathogenic SNVs in sperm or blood was much less numerous than that of BSS variants (Figures S1 and S2).

### Missense mutation was the dominant type

To advance this research, we analysed Different mutation types. As visualized in Figure 1(B) provides comparison of mutation types (nonsense mutation, stoploss, splicing, synonymous/nonsynonymous SNV) between sperm and blood of sperm donors and Figure 1(C) provides Indels (frameshift deletion/insertion, non-frameshift deletion/insertion) comparison between sperm and blood of sperm donors, the sperm and blood results within donor were consistent, with the most missense mutations (43), 30 indels, and 21 nonsense mutations (Figure 1(B)). From the indel point of view, there were 16 frameshift deletions, 10 frameshift insertions, and four non-frameshift deletions (Figure 1(C)).

### No pathogenic CNV was identified in semen donors

Table 2 depicts pathogenicity and CNV types in 40 sperm donors. A total of 328 CNVs (ranged from 0.2 Mb to5.8 Mb), were identified in these 40 semen donors; however, none of them was pathogenic. It was found that 164/328 CNVs (82 types each) were present in sperm as well as blood. Of these 82 CNV types, 16 were deletions and 66 were duplications. Of the other 164/328 CNVs, 122 CNVs were BDO, 42 CNVs were SDO (Table 2). Based on Supplementary Table S6 compares of all the non-pathogenic CNVs between semen and blood, the most frequent duplication was dup (chr17:44407503-44627544 (overlapped region)), and this region included the *ARL17B, NSFP1* and *LRRC37A2*. While the most frequent deletion was del (chr2:89975517-90260520 (overlapped region)), and this region included *IGKV2D-28* (Supplementary Table S6). CNVs found in blood were most prevalent in Chr17 (36), followed by Chr15 (21), while none were found in Chr12 or ChrY. Among the CNVs detected in sperm, Chr17 had the most (32), followed by Chr16 (13); Chr3, Chr11, Chr12, Chr13, Chr18, and ChrY had none, relevant details listed in Supplementary Table S7represented of chromosome distribution of CNVs.

**Table 2.** Pathogenicity and CNV types in sperm donors (*n* = 40).

Total		Pathogenicity		Types	
**Blood specific**	169	Benign	113	Deletion	13
				Duplication	100
		VUS	56	Deletion	32
				Duplication	24
**Blood consistent with semen**	70 (35 pairs)	Benign	58	Deletion	18
				Duplication	40
		VUS	12	Deletion	6
				Duplication	6
**Semen specific**	89	Benign	79	Deletion	17
				Duplication	62
		VUS	10	Deletion	2
				Duplication	8

### Simulated matching results for blood are consistent with results for sperm

**Figure 2.**
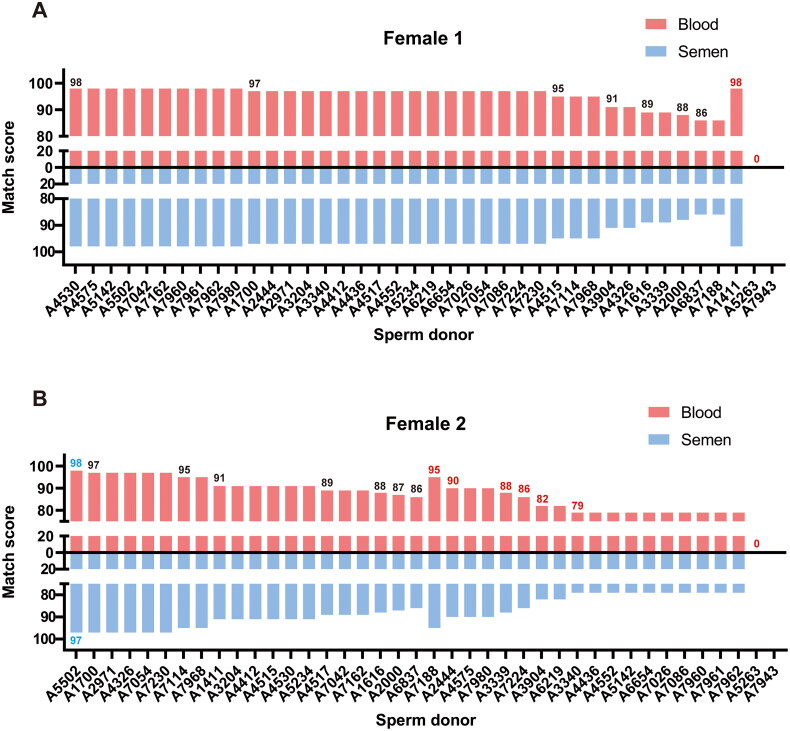
Simulated matching of blood and sperm with 2 women. Matching score of female 1/2 with 40 semen donors. The red bar marked was the not recommended donor.

Two women were randomly selected for simulated matching testing: a healthy woman (female 1) without pathogenic variants and another (female 2) who was an autosomal recessive carrier of oculocutaneous albinism (TYR (NM000372.5) c.896G > A p.Arg299His). Exome sequencing (ES) results were matched and scored against sperm and blood samples from 40 donors. For female 1, only three (7.5%) semen donors were not recommended for matching, whereas for female 2, 20 (50%) semen donors were not recommended. For female 1, recommended semen donors had a mean score of 95.5 and a maximum score of 98 (achieved by 10 semen donors). For female 2, recommended semen donors had a mean score of 92.3, with only one donor achieving the maximum score of 98. Matching scores between sperm and blood samples were nearly identical for female 1 across all 40 semen donors. However, a slight discrepancy was observed for female 2 with donor A5502, where the blood sample scored 98 and the sperm sample scored 97. This difference was primarily due to the detection of a heterozygous variant of uncertain significance (VUS) in the SUFU gene (NM016169.4: c.40G > C) in the sperm of A5502 donor, which was absent in his blood sample but present in female 2. Consequently, the sperm sample yielded a marginally lower matching score than the blood sample. Relevant details are documented in Figure 2 presents simulated matching of blood and sperm with two women.

## Discussion

Bell et al. conducted the initial carrier screening utilizing NGS, examining 448 severe recessive childhood diseases, revealing an average genomic carrier burden of 2.8 [18]. In 2015, ES was first implemented as a valuable genetic screening method for infertile couples undergoing assisted reproductive technology (ART). Analysis of the clinical dataset, comprising 2,161 samples, revealed that 84% were positive for genetic variants, with an average carrier burden of 2.3 variants per sample [19]. A previous study conducted exome sequencing (ES) on 43 semen donors, revealing that 90.7% tested positive for genetic variants, with an average carrier burden of 2.58 variants per donor. [4]. In our study, we conducted ES on sperm and blood samples obtained from 40 Chinese semen donors, revealing an average of 2.7 variants per donor. Despite the limited sample size, the observed variant count per sample aligns with findings from prior research studies [4]. In China, regulations permit a single semen donor to contribute to pregnancies for up to five women. If a donor carries genetic diseases, this significantly increases the risk of inherited disorders in the offspring of multiple families. Therefore, the screening of genetic disorders in semen donors is required to avoid scattering genetic diseases in the recipient population.

Although multiple facilities have conducted carrier screening using designed panels for different populations, and ACMG (American College of Medical Genetics) [20] and ACOG (American College of Obstetricians and Gynecologists) [21] have provided recommendations and guidelines for carrier screening, based on data from the US and EU populations, Chinese semen donors have been the subject of few carrier screening studies [4]. Detecting carrier frequencies in carrier screening is strongly influenced by the panel selection. The carrier frequencies for 415 genes connected to severe hereditary diseases in six varied ancestries were estimated by Guo and Gregg using an exome sequencing database. Ashkenazi Jewish carriers had up to 62.9% cumulative carrier rates, while East Asian carriers had 32.6% [22]. Meanwhile, data from the Translational Medicine Center at Fudan University Children’s Hospital demonstrated greater concordance with East Asian populations in the Exome Aggregation Consortium (ExAC) database compared to European cohorts [23]. This observed variability in allele frequencies across different ethnic groups underscores the importance of population-specific considerations in genetic screening and counselling practices; hence, genetic screening panels utilized in other nations may not be relevant to China. The ES method is widely utilized for targeted enrichment, mainly for its ability to efficiently sequence all exons in the human genome, making it ideal for studying monogenic disorders. The exome is believed to contain around 85% of mutations that have substantial effects on disease-related traits [24], necessitating only about 2% of the sequencing load in comparison to whole-genome sequencing (WGS). Despite its capability to accurately pinpoint SNVs and small InDels, ES requires extensive sequencing depth and meticulous data interpretation to detect large InDels, CNVs, and genomic rearrangements, aspects that are often not incorporated into the conventional analysis workflows of many projects. At present, ES combined with a panel screening is a potential and suitable option for semen donors for genetic assessment.

As we mainly performed the analysis by ES, the variants found were mainly located in exonic regions and dominated by point mutations. There might also be some specific structural variations that are not detected. In our study, we identified GJB2 variants as the predominant pathogenic mutations among semen donors, surpassing the carrier frequencies reported in previous studies [25,26]. To date, a total of 453 *GJB2* variants have been identified, of which more than 380 variants have been associated with deafness. Variants of GJB2 varied by ethnicity and region, and the c.35delG variant was mostly found in European and Middle Eastern populations [27,28]; the p.W24X variant was mainly distributed in India [29]; the c.167delT and p.R143W could be found in Jewish and Ghanaian populations, respectively [30,31].

While the c.235delC variant often appeared in East Asian populations [32], and one semen donor was also found carrying this variant in our study. In particular, 8 semen donors carried the c.109G > A (p.Val37Ile) variant, a higher proportion (8/40, 20%) than we expected, although this variant was known to be relatively common in Asian, especially Southeast Asian populations [33,34]. As GJB2 variants were diverse and some were non-penetrant, and genotypes were associated with the penetrance time of hearing impairment, variants in GJB2 could present as delayed and progressive deafness after birth [34–36]. Analysis revealed that among individuals with homozygous variants at the GJB2 locus (c.109G > A p.Val37Ile), 65% (11/17) experienced congenital hearing loss, while 35% (6/17) developed delayed hearing loss [37]. According to a 2012 epidemiological study, the p.Val37Ile variant, whether compound heterozygous or homozygous, resulted in progressive, delayed hearing loss [38]. Therefore, the high-frequency pathogenic variant carriage of GJB2 in semen donors is a problem that cannot be ignored.

We found that 7.5% of qualified semen donors were carriers of pathogenic variants (NM_000492.4: c.1210-12T [5] /c.1210-34TG [12] , c.1210-12T [5] /c.1210-34TG [13]) of *CFTR. CFTR* variants was the main pathogenic cause of congenital bilateral absence of the vas deferens (CBAVD) and cystic fibrosis (CF) [39]. 5 T is the most common mild *CFTR* variant, and the frequency of 5 T in CBAVD patients is approximately 0.25 in a pooled 38 CBAVD independent studies, and this variant is also prevalent in the general population at a frequency of approximately 0.05 [40]. Together with its adjacent TG repeat, poly-T impacts splicing efficiency of exon 10 of CFTR [41]. The combination of the 5 T variant with a severe CFTR mutation on another chromosome is the main cause of CBAVD [42]. Although incomplete penetrance of 5 T variant has also been reported [43,44], and CF is the least common inheritable disease in Asians [45]. The frequency of CFTR variants should be a concern in semen donors in China.

Among 40 semen donors, we found two patients with pathogenic variants of AD disease, corresponding to genes TPM2 and ACTN1, but both had no clinical manifestations. TPM2, associated with muscle contractility and mutations, can lead to muscle-related disorders without immediate clinical manifestations, suggesting variable expressivity. ACTN1 involves platelet functions, with mutations potentially causing mild thrombocytopenia with minimal clinical impact. As *TPM2* caused distal arthrogryposis is a heterogeneous category of inherited limb malformation syndromes with substantial clinical and genetic heterogeneity and variable expressivity [46–48]. This might explain why this semen donor had no apparent clinical manifestations. The ACTN1 gene, which encodes the actin-crosslinking protein α-actinin, is situated on chromosome 14q24.1. This gene comprises 21 exons spanning approximately 3.78 kb. α-Actinin 1 plays a crucial role in cytoskeleton organization, serving as an anchor for actin to various intracellular structures. It is predominantly expressed in platelets and megakaryocytes [49,50]. The mutation in ACTN1 is associated with a 50% reduction in platelet counts and a 30% increase in platelet size [51,52]. It has been reported that bleeding in *ACTN1*-related thrombocytopenia was absent or mild [53]. Thus, semen donors carrying pathogenic variants might not show clinical symptoms or show subclinical manifestations of disease.

Although it was postulated that the externalization of the testes was an evolutionarily conserved mechanism to restrict mutagenesis [54], all 108 pathogenic variants detected were consistently found in blood and sperm within the same patient, suggesting that all those variants might originate in embryonic development and occurred prior to primordial germ cell specification, and were stable throughout life. Nevertheless, low-frequency variants as well as non-pathogenic SNVs that are unique to blood (BDO) or sperm (SDO) were also found during data analysis. Our results showed that there were more non-pathogenic variants in sperm overall than in blood (Figure S1 and Figure S2). However, the non-pathogenic variant number of BDO or SDO was relatively small, when compared with non-pathogenic BSS variants. Previously, blood, saliva and oral samples from four unrelated individuals were compared by WGS. The data quality of different DNA sources was significantly different [55]. The accuracy difference in detecting short insertions and deletions was negligible. However, some saliva and oral samples exhibited a higher false positive rate (FPR). Moreover, CNV detection varied among different sample types from the same individual. Blood samples demonstrated sensitivity up to 25%, while saliva and oral samples with higher bacterial concentrations showed poor sensitivity [55]. Detection of CNV in blood-derived DNA is more sensitive than in unenriched saliva and oral samples, and the magnitude of the effect depends on the size and type of CNV [55]. Scheinfeldt et al. compared the mutation consistency between blood and blood lymphoblastoid cell line (LCL) cells of three generations and found that SNP consistency was good between whole blood DNA and LCL DNA of passage, while CNV consistency may decrease with the increase of passage [56]. In our analysis of sperm and blood samples from semen donors, we observed a higher prevalence of CNVs in blood compared to sperm, despite no significant difference in data quality. Notably, there is a current lack of published reports comparing SNVs and CNVs between sperm and blood samples. Theoretically, due to the increased number of cell divisions undergone by sperm, one would expect a higher frequency of variants in sperm samples. [57,58]. The results of SNV part were indeed the same, but blood had more CNVs. There was also a situation where, based on current sample types and testing methods, we had no way to rule out the effects of mosaicism, such as three sperm mosaicism types [59]. Although bulk sequencing could be used to detect male gonadal mosaicism, only type III mosaic mutations could reach the allele fraction of sperm samples (AF > 0.01) and be detected by this way [59]. Regarding the type III mutations detected in the sperm in some studies, between 33% and 50% are evident in the blood, saliva, or both [16,59]. Considering all the SNVs and CNVs with inconsistencies were non-pathogenic and low proportion mosaicism has a very low recurrence risk in offspring, we believe blood can be used to perform genetic screening on semen donors.

## Conclusions

We performed ES and panel analysis in sperm and blood samples from 40 Chinese semen donors, and the results of pathogenic variants were consistent between both samples, and no pathogenic CNV was detected in either sample. The matching results of sperm and blood were also consistent through simulated sperm donation matching tests performed by two women. Therefore, we believe that it is reasonable to choose either blood or sperm for genetic screening in semen donors.

## Supplementary Material

Supplemental Material

## Data Availability

The datasets and material used or analysed during the current study are available from the corresponding author on reasonable request.
