# Supplementary material for: Comparative genetic analysis of blood and semen samples in sperm donors from Hunan, China
Source: Ann Med. 2025 Jan 6;57(1):2447421. doi: 10.1080/07853890.2024.2447421 (PMC11721621; doi:10.1080/07853890.2024.2447421)
Supplement: Supplemental Material [file IANN_A_2447421_SM2148.zip › suppl_data/Figure Caption.docx]

**Supplementary Figure 1**. **The Benign/ Likely Benign variants of sperm and blood.** The B/LB variants of semen and blood from 40 sperm donors were divided into 12 categories according to the type of variation: stop-gain, stop-loss, splicing, 5′-UTR, 3′- UTR, UTR5; UTR3 (the same site was located in the 5′-UTR and 3′-UTR in different genes, respectively), nonsynonymous SNV, synonymous SNV, frameshift insertion/deletion, non-frameshift insertion/deletion. The median value of BDO (blood detectable only), BSS (blood-sperm shared) and SDO (sperm detectable only) is as indicated. Among all types of variants, the number of BSS variants was significantly higher than that of BDO or SDO (p < 0.001). *, p<0.01, **, p <0.01.

**Supplementary Figure 2. Variant of uncertain significance in 40 sperm donors’ semen and blood.** Variants of uncertain significance (VUSs) of sperm and blood from 40 sperm donors were divided into 12 categories according to the type of variation: stop-gain, stop-loss, splicing, 5′-UTR, 3′-UTR, UTR5; UTR3 (the same site was located in the 5′-UTR and 3′-UTR in different genes, respectively), nonsynonymous SNV, synonymous SNV, frameshift insertion/ deletion, non-frameshift insertion/deletion. The median value of BDO (blood detectable only), BSS (blood-sperm shared) and SDO (sperm detectable only) is as indicated. Among all types of variants, the number of BSS variants was significantly higher than that of BDO or SDO (p < 0.001). *, p<0.01. **, p <0.01.
