# Supplementary material for: Comparative genetic analysis of blood and semen samples in sperm donors from Hunan, China
Source: Ann Med. 2025 Jan 6;57(1):2447421. doi: 10.1080/07853890.2024.2447421 (PMC11721621; doi:10.1080/07853890.2024.2447421)
Supplement: Supplemental Material [file IANN_A_2447421_SM2148.zip › suppl_data/Table S1 revised.docx]

**Table S1 summary of the key guidelines and standards for sperm donor screening in China.**

| **Requirement/Condition** | **Description/Standard** |
| --- | --- |
| Age Range | Between 20 and 45 years old. |
| Health Status | Donors must be in good health, based on a physical examination and psychological evaluation. No familial history of genetic diseases. |
| Fresh Semen Analysis | Liquefaction time: less than 60 minutes;  Sperm concentration: more than or equal to 60 million per mL;  Progressive sperm motility: more than or equal to 60%;  Normal morphology: more than 30%. |
| Post-Thaw Semen Quality | Progressive sperm motility: more than or equal to 40%;  More than or equal to 12 million motile sperm per vial;  Frozen-thaw survival rate: more than or equal to 60%. |
| Laboratory Testing | HIV-1 and -2, hepatitis B and C, syphilis, gonorrhea, mycoplasma, chlamydia, cytomegalovirus, Toxoplasma gondii, rubella virus, herpes simplex virus types 1 and 2. Karyotype analysis is also conducted. |
| Quarantine Period for Samples | Minimum of 6 months followed by rescreening for HIV prior to use. |
