# Supplementary material for: Comparative genetic analysis of blood and semen samples in sperm donors from Hunan, China
Source: Ann Med. 2025 Jan 6;57(1):2447421. doi: 10.1080/07853890.2024.2447421 (PMC11721621; doi:10.1080/07853890.2024.2447421)
Supplement: Supplemental Material [file IANN_A_2447421_SM2148.zip › suppl_data/Table S2 revised.docx]

**Table S2. Demographic characteristics of 40 sperm donors**

| Characteristic | Mean±SD | Median(5^th^-95^th^%) |
| --- | --- | --- |
| Age(y) | 24.3±4.0 | 23(20-27) |
| Height(cm) | 164.9±1.7 | 165(162-167) |
| Weight(Kg) | 58.9±8.1 | 57(49-74) |
| Body mass index(kg/m^2^) | 21.7±2.9 | 21.1(17.7-27.0) |
