# Supplementary material for: Comparative genetic analysis of blood and semen samples in sperm donors from Hunan, China
Source: Ann Med. 2025 Jan 6;57(1):2447421. doi: 10.1080/07853890.2024.2447421 (PMC11721621; doi:10.1080/07853890.2024.2447421)
Supplement: Supplemental Material [file IANN_A_2447421_SM2148.zip › suppl_data/Table S7.docx]

**Supplementary Table S7. Chromosome distribution of CNVs**

| **Chromosome** | **Total CNVs** | **BSS** | **BDO** | **SDO** |
| --- | --- | --- | --- | --- |
| chr1 | 21 | 3 | 10 | 5 |
| chr2 | 18 | 1 | 12 | 4 |
| chr3 | 2 | 0 | 2 | 0 |
| chr4 | 10 | 0 | 9 | 1 |
| chr5 | 24 | 0 | 16 | 8 |
| chr6 | 6 | 0 | 5 | 1 |
| chr7 | 9 | 1 | 6 | 1 |
| chr8 | 22 | 4 | 8 | 6 |
| chr9 | 23 | 1 | 12 | 9 |
| chr10 | 10 | 1 | 7 | 1 |
| chr11 | 4 | 0 | 4 | 0 |
| chr12 | 0 | 0 | 0 | 0 |
| chr13 | 1 | 0 | 1 | 0 |
| chr14 | 19 | 1 | 9 | 8 |
| chr15 | 32 | 0 | 21 | 11 |
| chr16 | 30 | 5 | 12 | 8 |
| chr17 | 68 | 13 | 23 | 19 |
| chr18 | 2 | 0 | 2 | 0 |
| chr19 | 4 | 2 | 0 | 0 |
| chr20 | 3 | 0 | 2 | 1 |
| chr21 | 8 | 0 | 5 | 3 |
| chr22 | 10 | 2 | 3 | 3 |
| chrX | 2 | 1 | 0 | 0 |
| chrY | 0 | 0 | 0 | 0 |

BSS, blood-sperm shared; BDO, blood-detectable only; SDO, sperm-detectable only.
