# Supplementary figures and images for: Comparative genetic analysis of blood and semen samples in sperm donors from Hunan, China
Source: Ann Med. 2025 Jan 6;57(1):2447421. doi: 10.1080/07853890.2024.2447421 (PMC11721621; doi:10.1080/07853890.2024.2447421)

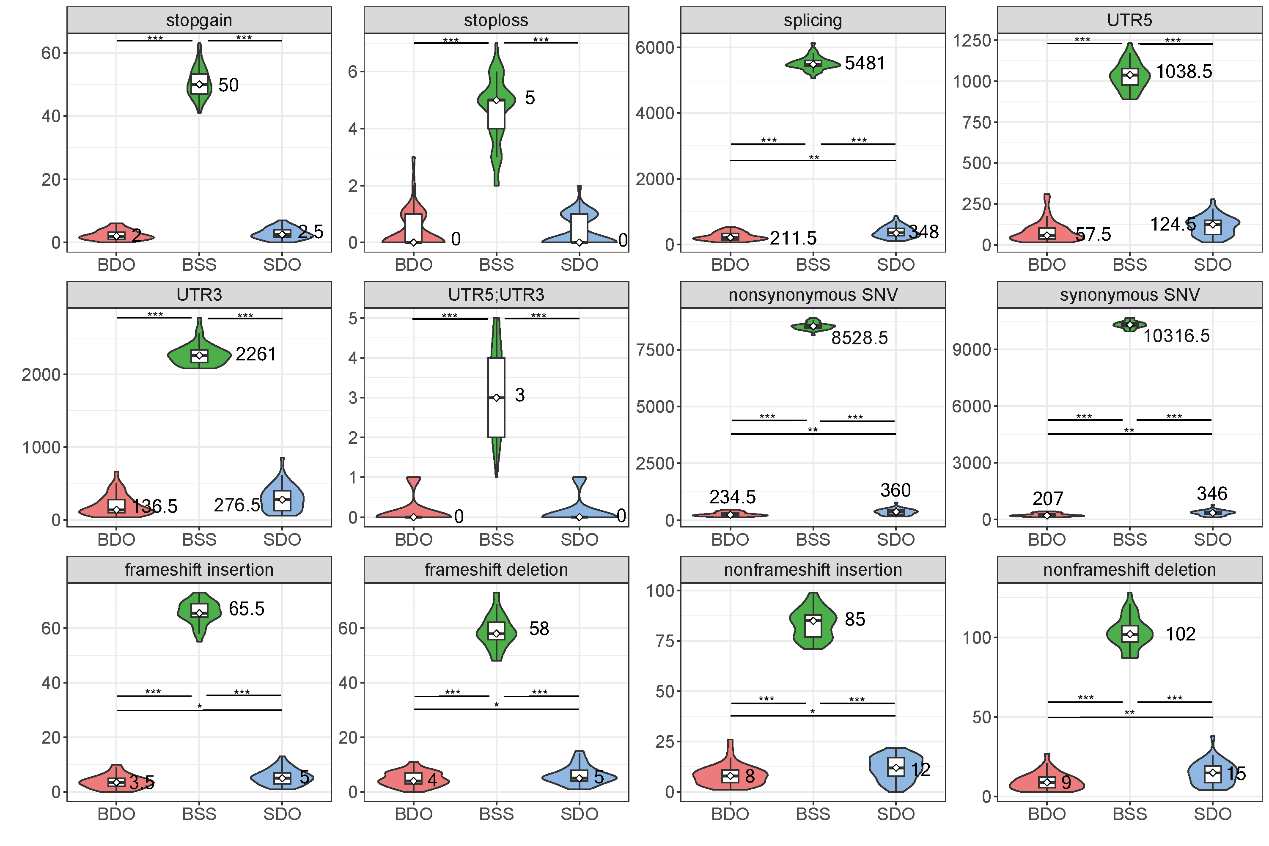

Supplement: Supplemental Material [file IANN_A_2447421_SM2148.zip › suppl_data/FigureS1.tif]

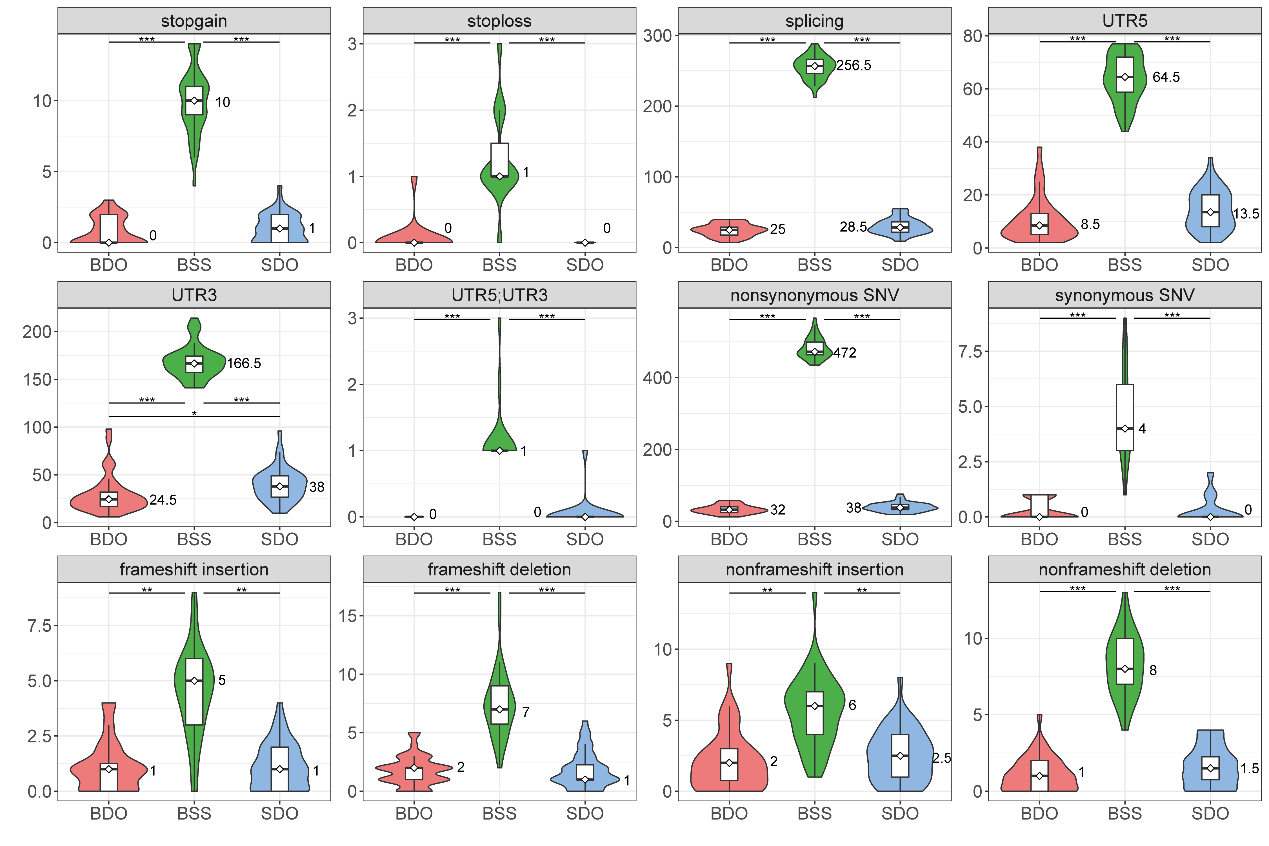

Supplement: Supplemental Material [file IANN_A_2447421_SM2148.zip › suppl_data/FigureS2.tif]
